# *Faecalibacterium prausnitzii* Skews Human DC to Prime IL10-Producing T Cells Through TLR2/6/JNK Signaling and IL-10, IL-27, CD39, and IDO-1 Induction

**DOI:** 10.3389/fimmu.2019.00143

**Published:** 2019-02-06

**Authors:** Joudy Alameddine, Emmanuelle Godefroy, Loukas Papargyris, Guillaume Sarrabayrouse, Julie Tabiasco, Chantal Bridonneau, Karina Yazdanbakhsh, Harry Sokol, Frédéric Altare, Francine Jotereau

**Affiliations:** ^1^CRCINA, INSERM, Université d'Angers, Université de Nantes, Nantes, France; ^2^CRCINA, INSERM, Université de Nantes, Université d'Angers, Angers, France; ^3^LabEx IGO “Immunotherapy, Graft, Oncology”, Angers, France; ^4^Vall d'Hebron Institute of Research, Barcelona, Spain; ^5^Micalis Institute, INRA, AgroParisTech, Université Paris-Saclay, Jouy-en-Josas, France; ^6^New York Blood Center, LFK Research Institute, New York, NY, United States; ^7^APHP Laboratoire des Biomolécules (LBM), CNRS, INSERM, Sorbonne Universités, Paris, France; ^8^Laboratoire des Biomolécules, Département de Chimie, CNRS, PSL Research University, Paris, France

**Keywords:** gut microbiota, Tr1-like Treg, TLR2/6, JNK, CD39, IDO-1, dendritic cells, colon homeostasis

## Abstract

The human colonic mucosa contains regulatory type 1-like (Tr1-like, i.e., IL-10-secreting and Foxp3-negative) T cells specific for the gut *Clostridium Faecalibacterium prausnitzii (F. prausnitzii)*, which are both decreased in Crohn's disease patients. These data, together with the demonstration, in mice, that colonic regulatory T cells (Treg) induced by *Clostridium* bacteria are key players in colon homeostasis, support a similar role for *F. prausnitzii*-specific Treg in the human colon. Here we assessed the mechanisms whereby *F. prausnitzii* induces human colonic Treg. We demonstrated that *F. prausnitzii*, but not related Clostridia, skewed human dendritic cells to prime IL-10-secreting T cells. Accordingly, *F. prausnitzii* induced dendritic cells to express a unique array of potent Tr1/Treg polarizing molecules: IL-10, IL-27, CD39, IDO-1, and PDL-1 and, following TLR4 stimulation, inhibited their up-regulation of costimulation molecules as well as their production of pro-inflammatory cytokines IL-12 (p35 and p40) and TNFα. We further showed that these potent tolerogenic effects relied on *F. prausnitzii*-induced TLR2/6 triggering, JNK signaling and CD39 ectonucleotidase activity, which was induced by IDO-1 and IL-27. These data, together with the presence of *F. prausnitzii*-specific Tr1-like Treg in the human colon, point out to dendritic cells polarization by *F. prausnitzii* as the first described cellular mechanism whereby the microbiota composition may affect human colon homeostasis. Identification of *F. prausnitzii*-induced mediators involved in Tr1-like Treg induction by dendritic cells opens therapeutic avenues for the treatment of inflammatory bowel diseases.

## Introduction

Regulatory T cells (Treg) induced locally by the microbiota contribute to gut homeostasis (1, 2). In mice, a panel of 13 *Clostridium* IV and XIVa bacteria plays a major role in this process by inducing colonic Foxp-3 Treg that prevent colitis and allergy (1). However, in the human colon microbiota, induced-Foxp3 Treg have not been reported so far. Instead, we have shown that type-1 like (Tr1-like, i.e., IL-10-secreting, Foxp3-negative) Treg, characterized by a double positive CD4CD8α (DP8α) phenotype, are abundant in the healthy human colon, circulate in blood, and are decreased in inflammatory bowel disease (IBD) patients in both compartments. Strickingly, suggesting that DP8α Treg could be functional homologs of mouse colonic Foxp3 Treg, we established their specificity for the *Clostridium* IV *Faecalibacterium prausnitzii* (*F. prausnitzii*), one of the most abundant commensal in the microbiota of healthy subjects (3, 4). Interestingly, this bacterium is decreased in IBD patients (5–7) and exhibit anti-inflammatory properties, suggesting its role in gut homeostasis (5, 8–10). Altogether, these previous data clearly document *F. prausnitzii* contribution to the induction of human colonic Tr1-like Treg and support a role for these Treg in colon homeostasis.

Specialized dendritic cells (DC) play a pivotal role in tissue homeostasis by limiting effector T cells and promoting the differentiation of Treg: Foxp3 or Tr1-like (11, 12). In the mouse gut, induction of these regulatory DC depends on local factors such as diet antigens, retinoic acid, and TGF-β, in the small intestine (2, 11), or on commensal bacteria, especially *Clostridium* and their metabolite butyrate, in the colon (1, 2, 13). Moreover, some of the mediators whereby tissue DC induce Treg have been identified, among which regulatory cytokines, especially TGF-β and IL-10 regarding Foxp3 Treg induction (11, 12, 14) or IL-10 and IL-27 for the induction of Tr1-like Treg (15–17), as well as immunoregulatory molecules such as the tryptophan catabolizing enzyme indoleamine 2,3 dioxygenase (IDO1) (11), heme-oxygenase-1 (HO-1) (18), retinoic acid (2, 19), PDL-1 (20) and the ectonucleoside triphosphate diphosphohydrolase 1 (ENTPD1 or CD39) (21), the latter being induced on DC by IL-27 (22).

Besides *Clostridium*, other gut bacteria, especially *Bacteroides fragilis, via* its carbohydrate antigen PSA (23, 24), *Bifidobacterium breve* (25), and specific *Lactobacilli* (26) may promote the differentiation of Foxp3 or Tr1 Treg *in vitro* or in mice through DC modulation. However, the physiological relevance of these results remains unclear.

Here we asked whether *F. prausnitzii* could induce human colonic Tr1-like Treg through modulating DC function. Human monocyte-derived and myeloid DC were exposed to *F. prausnitzii* or to related bacteria, *Clostridium ramosum* and *Clostridium symbiosum*, that induce Foxp3 Treg in mice. DC were then analyzed for their phenotype, cytokine secretion, T cell priming abilities as well as for the mechanisms involved in *F. prausnitzii*-mediated DC alterations.

## Methods

### Antibodies and Reagents

We used: PE-labeled antibodies to CD40 (clone 5C3), CD80 (clone L307.4), CD83 (clone HB15e), CD39 (clone TU66), PDL-1 (clone MIH1), as well as anti-CD4-APC (clone SK3) all from Becton Dickinson: (BD), anti-CD86-PE (clone HA5.2B7, Beckman Coulter), anti-IL-12p35/p70-PE (clone REA121, Miltenyi Biotech), anti-IL-12/IL-23p40-APC (clone C11.5, BioLegend), anti-IL-10-PE (clone JES3-19F1, BD), anti-IFN-γ-APC (clone B27, BD), anti-IL-13-PE (clone JES10-5A2, BD), anti-IDO-1-APC (clone 70083, R&D), anti-HO-1 (HO-1-1, Thermoscientific). For blocking experiments, we used rat neutralizing Ab to IL-10 (10 μg/ml, BD), IL-10R (10 μg/ml, R&D Systems), TLR2, TLR6, and Rat IgG control (20 μg/ml, InvivoGen), the JNK inhibitor SP600125 (10 μM, Abcam), the inhibitor of the CD39 ecto-nucleotidase activity Pom1 (10 μM, Tocris) and the IDO1 inhibitor (400 μM, Sigma Aldrich). DC were incubated with the inhibitor for 45 min before bacteria addition.

### Bacteria

*Faecalibacterium prausnitzii* A2-165, (*F. prausnitzii), Clostridum ramosum* DSM1402, (M88), and *Clostridium symbiosium* DSM934, (M89), were grown as described elsewhere (3). The number of bacteria was determined using the conversion factor 1.3 × 10^8^ bacteria/ml/OD_600_ unit. Bacteria were pelleted from the culture and sonicated.

### Generation of DC

Peripheral Blood samples were obtained from healthy volunteers who gave informed consent, at the Etablissement Français du Sang (EFS, Pays de Loire, France). The study was approved by the committee for Research Ethics concerning human subjects: Convention CPDL-PLER-2018 021. Research was carried out in accordance with the declaration of Helsinki. Monocytes were purified from PBMC using CD14 microbeads (Miltenyi Biotec) and were differentiated into DC by a 4 to 5 day-culture with rhGM-CSF (500 IU/ml) and rhIL-4 (300 IU/ml) (CellGenix). Non-adherent cells were harvested, counted and distributed in fresh GM-CSF/IL-4-containing medium together or not with the bacteria for 24–48 h following or not 45 min incubation with an inhibitor or its vehicle. In some experiments, day-5 DC were incubated for 24 h with rhIL-27 (100–200 ng/ml, PeproTech). In some experiments DC were exposed to the bacteria at day 0 of the culture. No difference was observed between DC obtained in this condition and DC exposed later to the bacteria. After exposure to the bacteria, day-5 to 6 DC were in some cases stimulated for 16–48 h (as indicated) by ultrapure LPS (200 ng/ml, InvivoGen) with or without rhIFN-γ (Miltenyi Biotec, 1,000 IU/ml) and/or R848 (5 μg/ml). LPS-stimulated DC adhered and were harvested following EDTA treatment.

### Isolation of Myeloid Dendritic Cells and CD4 T Cells

Myeloid CD1c BDCA-1+ DC were isolated from PBMCs using a selection kit (MACS Miltenyi Biotech). Naïve or total CD4 T cells were isolated from PBMCs using a selection kit (eBioscience & MACS Miltenyi Biotech, respectively).

### CD4 T Cell Priming and Responses

CD4 T cells were stained with Violet Proliferation Dye 450 (VPD) (BD Bioscience, 1 μM) and co-cultured with allogeneic DC (ratios 20:1 or 50:1) exposed or not to bacteria and then matured by LPS. After 10 days, CD4 T cells were re-stimulated with CD3/CD28-coated beads (Gibco) (ratio 3:1) for 6 h for IFN-γ and IL-13 staining or 24 h for IL-10 staining, in the presence of BFA for the last 5 h.

### Flow Cytometry

Cell surface staining was done at 4°C in PBS 0.1% BSA using specific Ab and their isotype control. Results are expressed as relative fluorescence intensity (RFI). For intracellular staining, cells were fixed in 4% paraformaldehyde for 10 min, and stained for 30 min at RT in permeabilization buffer (InvitroGen). Fluorescence was measured with a Canto II flow cytometer and analyzed using Diva software (BD).

### ELISA-Based Assays

The levels of IL-10, IL-12p70, TNF-α, IL-6 (eBioscience), active TGF-β1 (Biolegend), and IL-27 (R&D Systems) were determined in DC supernatants by specific sandwich ELISA, according to the manufacturer's guidelines.

### Statistics

Two-tailed Student's *t*-tests or Wilcoxon tests were performed for paired measurements with GraphPad Prism software. *p* < 0.05 were considered significant.

## Results

### *F. prausnitzii*-Exposed Dendritic Cells (DC) Prime IL-10-Secreting T Cells

**Figure 1:**
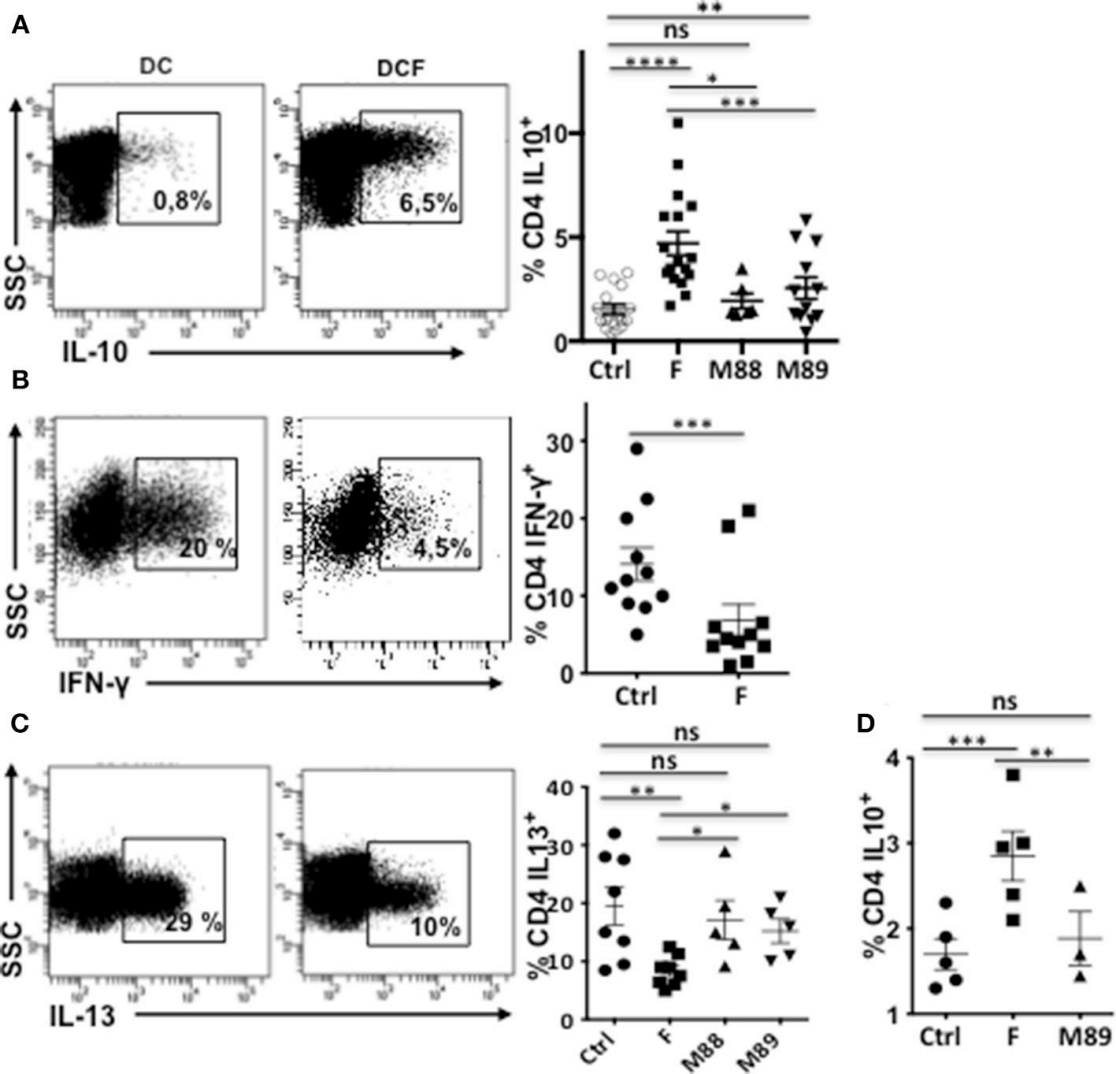
Exposure to *F. prausnitzii* (F) promotes DC ability to prime IL-10-secreting T cells. VPD-stained naïve CD4^+^ T cells were stimulated by LPS-stimulated DC, pre-exposed or not to indicated bacteria at day 0 [**(A)** *n* = 6–18, **(B)** *n* = 11, **(C)** *n* = 5–7] or from day 4 to 6 of their differentiation [**(D)** *n* = 5], and were re-stimulated by CD3/CD28 beads. Representative examples and mean percentages ± sem of CD4 T cells expressing indicated cytokines. Paired *t*-test: *p < 0.05, **p < 0.005, ***p < 0.0005, and ****p < 0.00005.

We first asked whether *F. prausnitzii* could specifically promote DC ability to prime IL-10-secreting T cells. VPD-stained naïve CD4 T cells were stimulated by allogeneic monocyte-derived DC exposed (DCF), or not (DC), to *F. prausnitzii* (or M88 or M89) at the beginning or at day 4 of their differentiation and stimulated or not by LPS. Ten days later, VPD^low^ T cells were tested for their ability to produce various cytokines. Clearly, T cells primed by DCF produced more IL-10 than those primed with untreated DC or DC exposed to other bacteria (Figure 1A). Moreover, DCF induced less IFN-γ-secreting T cells than untreated DC (Figure 1B) and less IL-13-secreting T cells than untreated DC and DC exposed to M88 or M89 (Figure 1C). Also, independently of LPS activation, DC exposed to *F. prausnitzii* for the last 48 h primed more IL-10-secreting CD4 T cells than untreated DC and M89-exposed DC (Figure 1D). Therefore, a single exposure to *F. prausnitzii*, but not to other bacteria, for at least 2 days up-regulated the ability of DC (stimulated or not by LPS) to prime CD4 T cells secreting IL-10, a cytokine profile compatible with that of *F. prausnitzii*-specific DP8α Treg (3, 4). Moreover, DCF induced a lower proliferation of allogeneic CD4 T cells than untreated DC did (Supplementary Figure S1A). We therefore studied the molecular basis behind *F. prausnitzii*-specific alteration of the T cell polarizing properties of DC.

### *F. prausnitzii* Induces or Up-Regulates the Expression of Tr1-Inducers by DC

**Figure 2:**
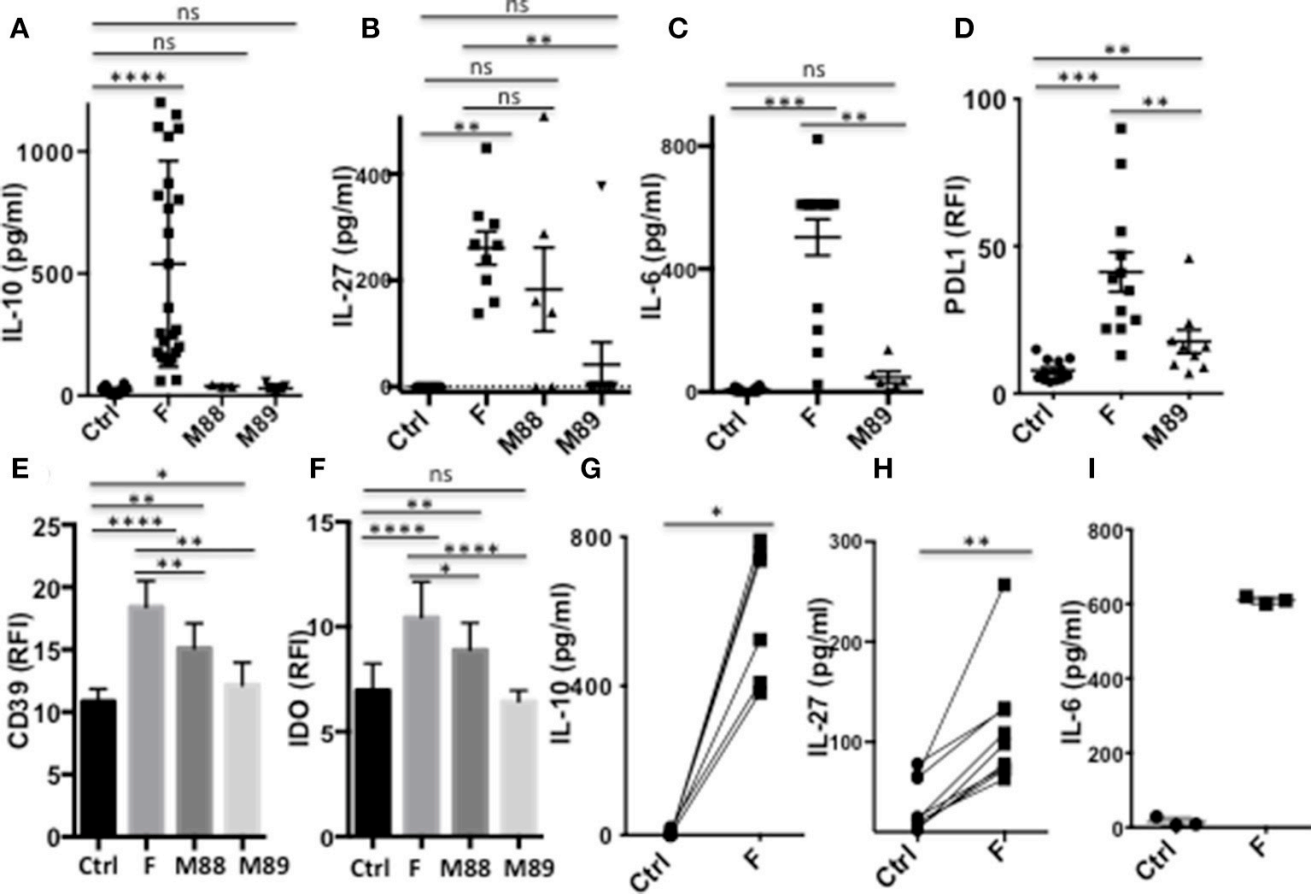
DC exposure to *F. prausnitzii* promotes the production of immunoregulatory molecules. **(A)**: IL-10 (Ctrl and F *n* = 26, M88 *n* = 3, M89 *n* = 9), **(B):** IL-27 (Ctrl, F and M89 *n* = 9, M88 *n* = 6) and **(C):** IL-6 (Ctrl and F *n* = 12, M89 *n* = 6) -secretion by DC exposed or not to indicated bacteria during the last 24 h. **(D–F)**: expression of PDL-1 and CD39 (*n* = 15–29) or IDO-1 (*n* = 18–25) by DC exposed or not to indicated bacteria for the last 24 or 48 h. **(G–I)**: IL-10 (*n* = 6), IL-27 (*n* = 9), and IL-6 (*n* = 3) secretion by myeloid DC maintained for 24 h in culture with or without *F. prausnitzii*. Results as mean ± sem. Wilcoxon test. *p < 0.05, **p < 0.005, ***p < 0.0005, ****p < 0.00005.

We first asked whether *F. prausnitzii* could induce DC to secrete regulatory cytokines. DCF, but not DC exposed to M88 or M89, secreted IL-10 (Figure 2A) in a dose-dependent manner (Supplementary Figure S2A). Similarly, *F. prausnitzii*-exposed DC systematically secreted IL-27 (Figure 2B and Supplementary Figure S2B), and IL-6 (Figure 2C), a cytokine produced by intestinal DC and involved in Tr1 cell generation (27, 28), while DC from only 4 out of 6 and 1 out of 9 donors secreted IL-27 upon exposure to M88 and M89, respectively and the latter bacterium did not induce IL-6 secretion (Figures 2B,C). In contrast, *F. prausnitzii*-exposed DC did not secrete active TGF-β1 (data not shown), a cytokine involved in Foxp3 Treg generation. Expression of additional immuno-regulatory molecules known to contribute to Tr1 and/or Foxp3 induction was then tested: strickingly, *F. prausnitzii*-exposed DC up-regulated PDL-1 (Figure 2D), CD39 (Figure 2E), and IDO1 (Figure 2F), but not HO-1 (Supplementary Figure S2C). In contrast, M89 poorly up-regulated, or not, IDO1 and CD39 and poorly induced PDL-1 (Figures 2D–F) and M88 increased the CD39 and IDO1 expression but to a lower extent than *F. prausnitzii* did (Figures 2E,F).

Importantly, purified myeloid DC exposed to *F. prausnitzii* for 24 h secreted IL-10, IL-27 and IL-6 (Figures 2G–I).

### *F. prausnitzii* Inhibits LPS-Induced Maturation of DC and Secretion of Pro-Inflammatory Cytokines

**Figure 3:**
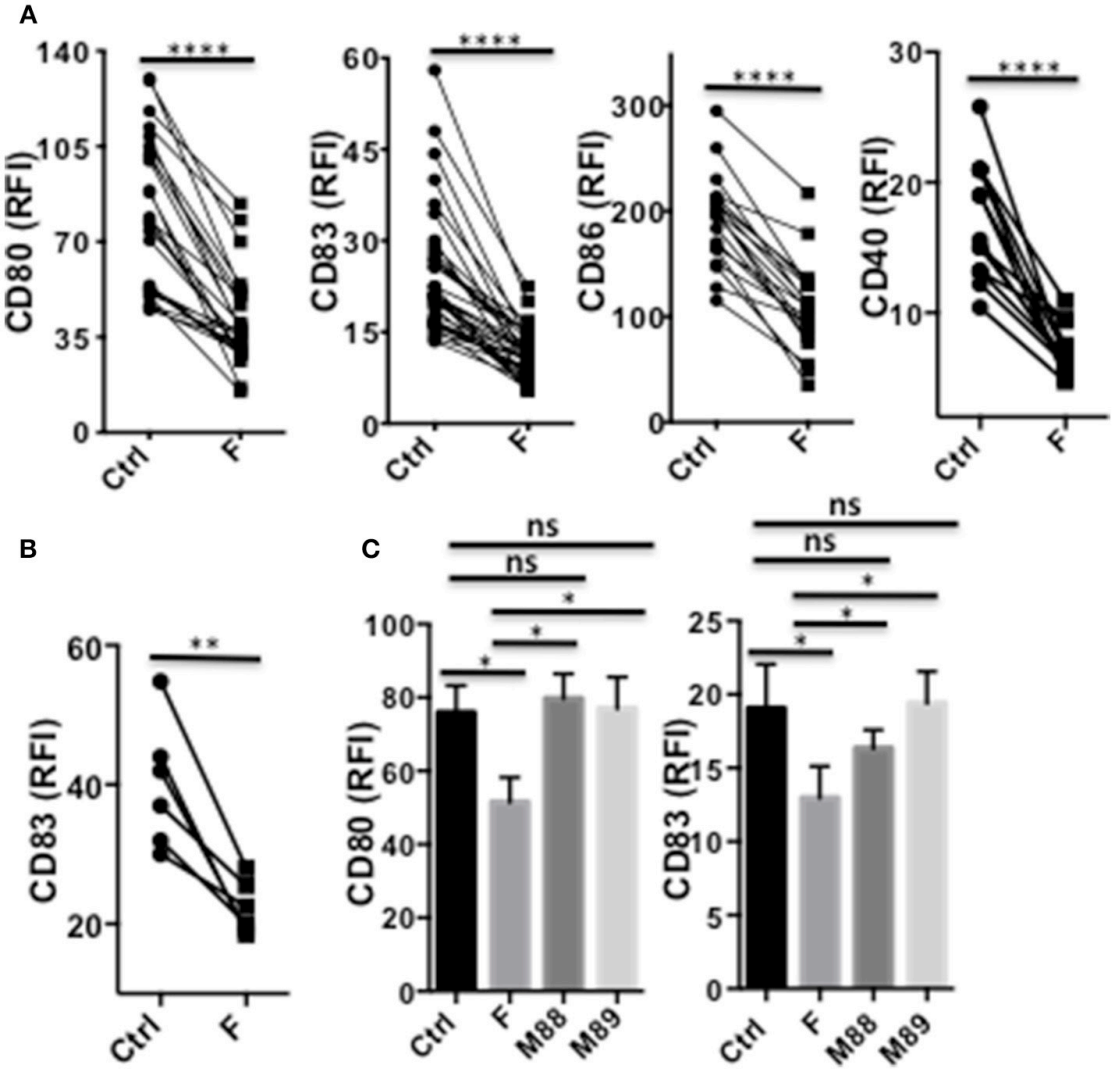
A single exposure to *F. prausnitzii* inhibits DC maturation. **(A)** Expression of CD80, CD83, CD86 or CD40 by DC exposed or not to *F. prausnitzii* at the beginning of their differentiation or **(B)** at day 5 of their differentiation (*n* = 6), and stimulated by LPS at day 6 during 48 h; **(C)** Expression of CD80 or CD83 (*n* = 7) by bacteria-exposed DC. Marker expression was measured by flow cytometry using a Canto II FACS Scan and expressed as relative fluorescence intensity (RFI) ± sem. Paired *t*-test. *p < 0.05, **p < 0.005, ****p < 0.00005.

In their immature or partially mature state, DC favors priming of IL-10-secreting Treg (29). We therefore assessed whether *F. prausnitzii* could alter TLR4-induced DC maturation. The expression levels of CD80, CD83, CD86, and CD40 remained significantly lower in DC exposed to *F. prausnitzii* (Figure 3A and Supplementary Figure S3A), even for only 24 h (Figure 3B), than in untreated DC. M88 and M89 Clostridia did not share such ability (Figure 3C).

**Figure 4:**
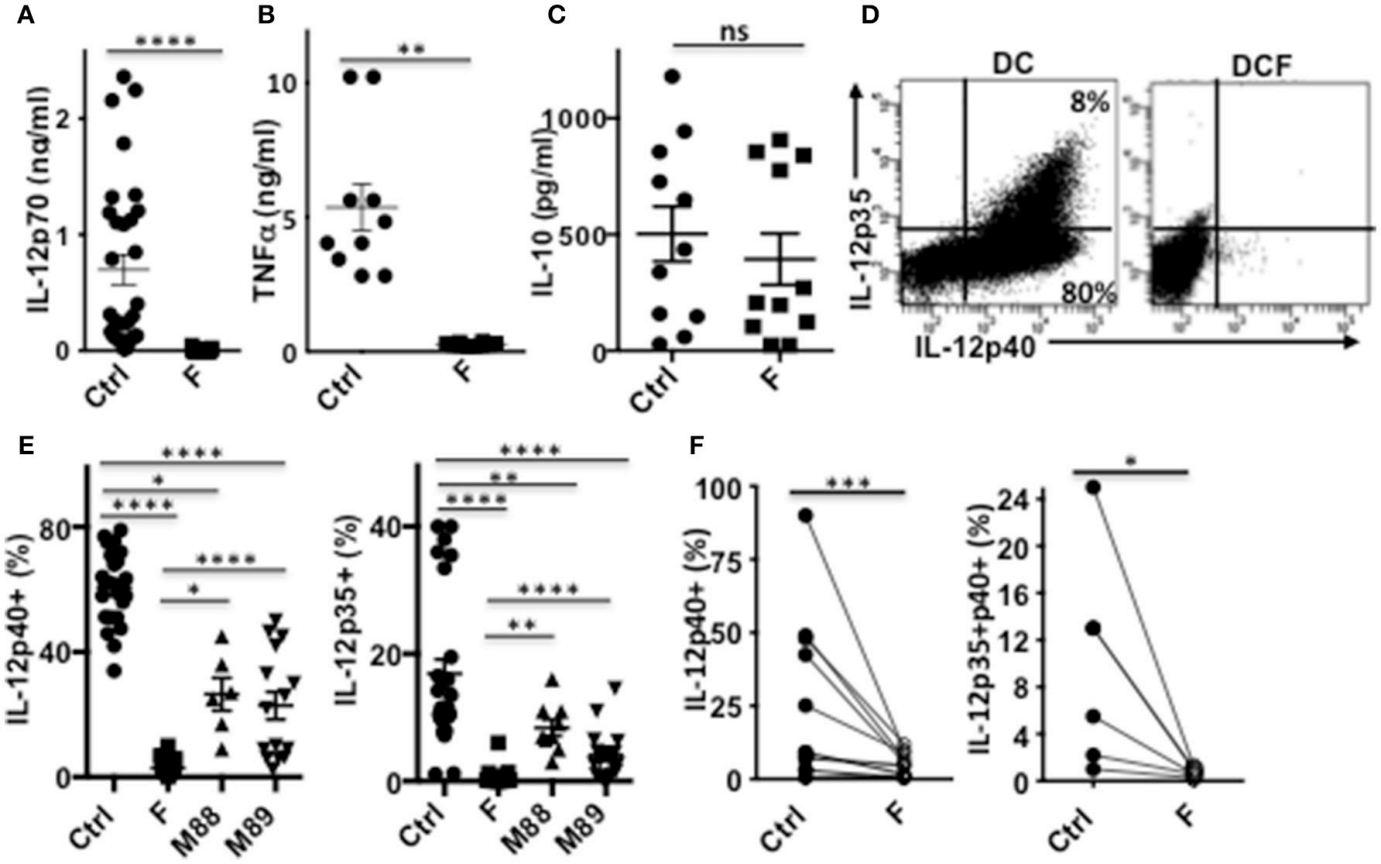
DC exposure to *F. prausnitzii* switches their cytokine profile from pro-inflammatory to anti-inflammatory. IL-12p70 **(A)**, TNF-a **(B)**, and IL-10 levels **(C)** secreted in response to LPS by DC exposed or not (Ctrl) to *Fprau* at the beginning of their differentiation. **(D)** Representative dot plot of intracellular IL-12p35 and p40 co-labeling in LPS-stimulated DC exposed or not to *F. prausnitzii*. **(E)** LPS-induced IL-12p40 (*n* = 6–24) and IL-12p35 (*n* = 9–27) expression by DC exposed or not (Ctrl) to indicated bacteria (ratio:1:1) during the last 48 h. **(F)** IL-12p40 (*n* = 12) and p35 (*n* = 6) expression by myeloid DC maintained for 24 h in culture with or without *F. prausnitzii*, and then stimulated 12 h with LPS. Wilcoxon test. *p < 0.05, **p < 0.005, ***p < 0.0005, ****p < 0.00005.

Strikingly, a single exposure to *F. prausnitzii* of DC totally blocked their ability to secrete IL-12p70 and TNF-α (Figures 4A,B), but did not alter their IL-10 secretion (Figure 4C), in response to LPS. Since IL-12p70 production relies on the induction of p35 and p40 subunits, which are regulated by distinct pathways, we then studied the expression of each chain using intracellular staining. Upon stimulation by LPS, LPS+IFN-γ or LPS+R848, the production of both chains was essentially abolished at the DC: *F. prausnitzii* ratio 1:1 in monocyte-derived DC (Figures 4D,E) as well as in myeloid DC (Figure 4F). As shown for the p40 subunit (Supplementary Figure S3B), inhibition of IL-12 chains by *F. prausnitzii* was dose-dependent and total at the *F. prausnitzii*:DC ratio 1:1. Unexpectedly, M88 and M89 also inhibited DC expression of IL-12p40 and p35 chains (Figure 4E). Nonetheless, these inhibitions did not completely abrogate IL-12p70 secretion, which was highly variable between donors (Supplementary Figure S3C), at variance with the complete inhibition observed in all DCF tested (Figure 4A).

### *F. prausnitzii* Modulates DC Functions Through TLR2/6 and JNK Signaling

We next studied which signaling pathways were responsible for *F. prausnitzii*-mediated alterations of DC functions. Several TLRs mediate activation of innate cells by bacteria. *F. prausnitzii* has been shown to lack TLR4 ligand, but to express TLR2 and TLR2/6 ligands (10). TLR2 has been linked to the tolerogenic roles of commensals, such as *Bacteroides fragilis* (23) and *Bifidobacterium breve* (25). TLR2/6 was shown to drive the induction by *Yersinia pestis* of IL-10-producing DC via the JNK MAP kinase-signaling and induction of Tr1-like Treg (30). Furthermore, TLR2/6 ligands can stimulate IL-27 production by DC (30, 31). We therefore assessed the impact on DCF of neutralizing TLR2 and TLR6 using specific antibodies (Abs), and JNK, via the SP600125 inhibitor.

**Figure 5:**
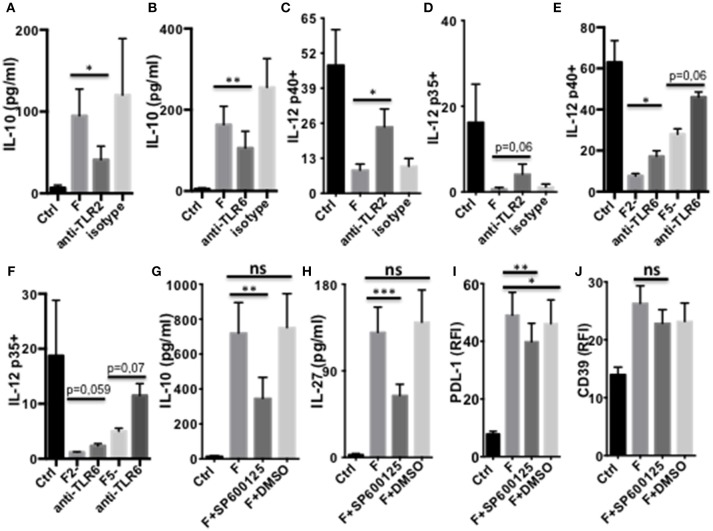
TLR2/6- and JNK-dependent modulation of DC function by *F. prausnitzii***. (A,B)*:***IL-10-secretion by d5-DC exposed or not to *F. prausnitzii* for the last 24 h, in the presence or not of neutralizing anti-TLR2 [**(A)** *n* = 3] or anti-TLR6 Ab [**(B)** *n* = 8]. **(C–F)**: LPS-induced IL-12p40 or p35 expression by DC exposed or not to *F. prausnitzii* at DC:bacterium ratios 1:1 (F or F1), 2:1 (F2-) or 5:1 (F5-), during the last 48 h in the presence or not of neutralizing anti-TLR2 [**(C)** *n* = 6, **(D)** *n* = 5] or -TLR6 Ab [**(E)** *n* = 3, **(F)** *n* = 3]. Secretion of IL-10 [**(G)** *n* = 9), IL-27 [**(H)** *n* = 14] or expression of PDL-1 [**(I)** *n* = 9] or CD39 [**(J)** *n* = 12] by DC exposed or not (Ctrl) to *F. prausnitzii* for the last 24 h, in the presence or not of the JNK inhibitor or its vehicle control DMSO. Paired *t*-test for *n* = 3, Wilcoxon test otherwise. *p < 0.05, **p < 0.005, ***p < 0.0005.

Both TLR2 and TLR6 inhibition decreased IL-10 secretion (Figures 5A,B) and partly restored LPS-induced expression of IL-12 chains in DCF (Figures 5C–F and Supplementary Figure S4A). Whether neutralization of these receptors altered IL-27 production and CD39 expression by DCF was not conclusive, since these responses were similarly inhibited by the specific Abs and their isotype (Supplementary Figures S4B,C). Inhibition of JNK, a MAPK activated by a TLR2/6 ligand (30), decreased the production of IL-10 and IL-27 (Figures 5G,H) and, to a lower extent, the expression of PDL-1 (Figure 5I) by DCF. In contrast, CD39 expression (Figure 5J) and IL-12 inhibition (Supplementary Figures S4E,F) remained unaffected by the JNK inhibitor, and its contribution to IDO1 expression was unclear due to a similar effect of the vehicle control (Supplementary Figure S4D)

### Role of IL-10, IL-27, CD39, and IDO1 in *F. prausnitzii*-Dependent Modulation of DC

**Figure 6:**
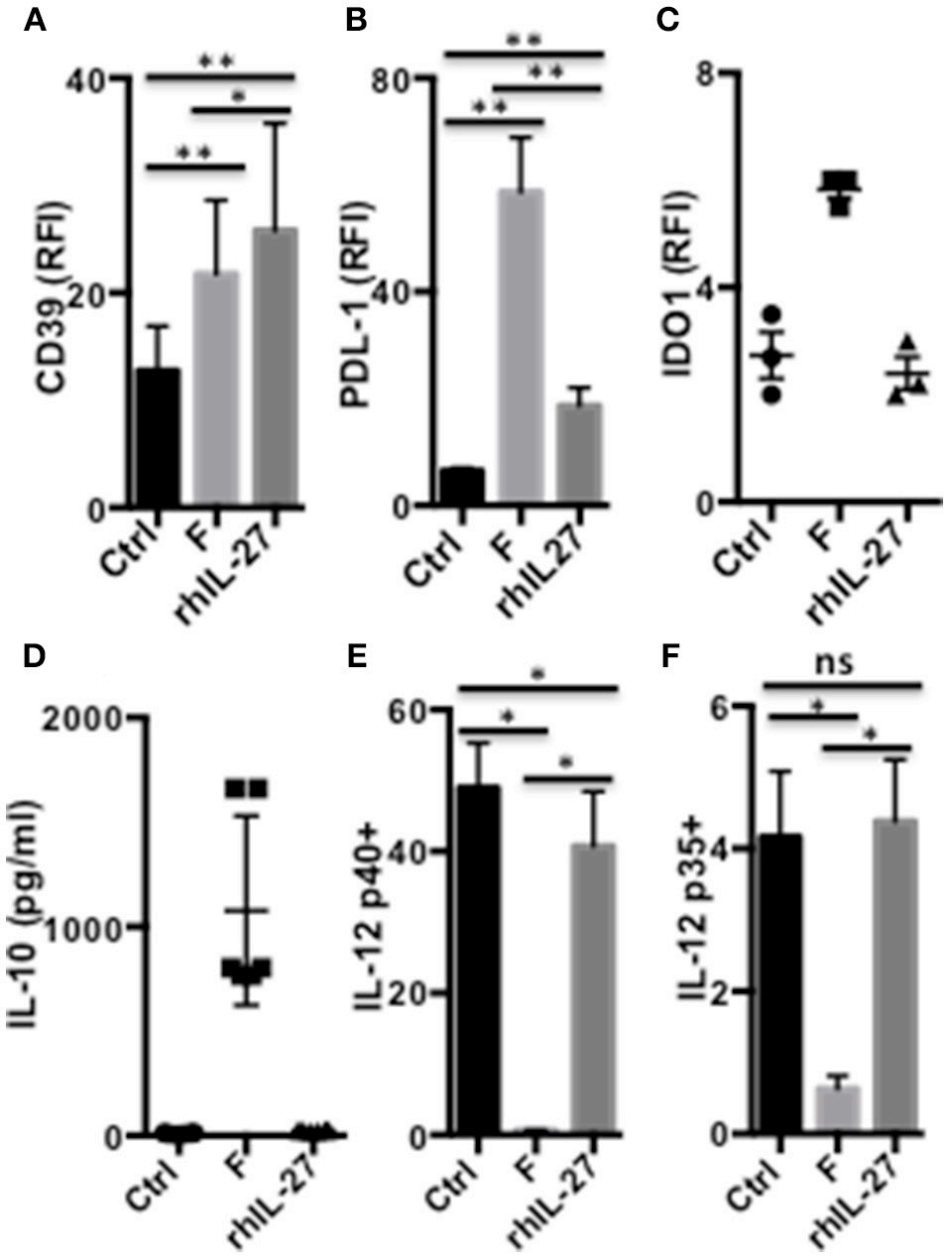
IL-27-dependent modulation of DC functions by *F. prausnitzii*. Expression of CD39 [**(A)** *n* = 8], PDL-1 **[(B)** *n* = 9] or IDO1 [**(C)** *n* = 3], and IL-10 secretion [**(D)** *n* = 6] by DC incubated or not for the last 24 h with *F. prausnitzii* or with rhIL-27. IL-12p40 [**(E)** *n* = 6] or p35 [**(F)** *n* = 6] expression by DC incubated or not for the last 24 h with *F. prausnitzii* or with rhIL-27 and then stimulated overnight by LPS. Wilcoxon test. *p < 0.05, **p < 0.005.

As shown above (Figure 2), a single *F. prausnitzii*-exposure induced DC to express IL-10, IL-27, CD39, IDO1, and PDL-1, molecules known to play major roles in Foxp3 or Tr1-Treg induction. To further understand the mechanisms leading to these functional alterations, we assessed the hierarchical expression of these molecules in DCF, using specific inhibitors and human recombinant IL-27 (rhIL-27). DC exposed to rhIL-27 up-regulated CD39, at a similar level than DCF did (Figure 6A), and PDL-1 to a lower extent (Figure 6B). In contrast, rhIL-27 did not induce IDO1 expression (Figure 6C), IL-10 secretion (Figure 6D), and did not inhibit LPS-induced IL-12p35 and p40 expression (Figures 6E,F). Therefore, IL-27 may mediate up-regulation of CD39 in DCF.

**Figure 7:**
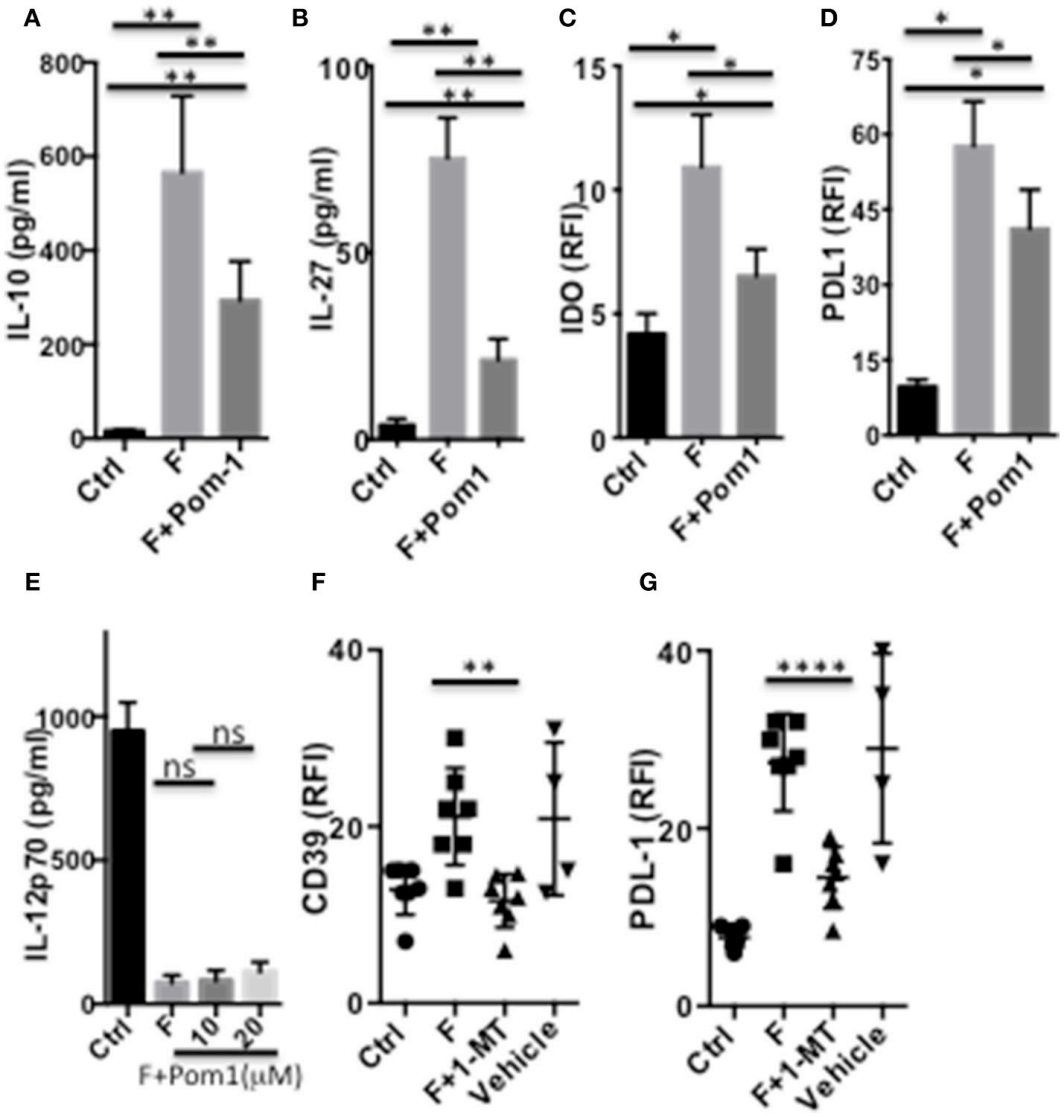
CD39 and IDO1-dependent modulation of DC functions by *F. prausnitzii*. Secretion of IL-10 [**(A)** *n* = 8] or IL-27 [**(B)** *n* = 10] or expression of IDO1 [**(C)** *n* = 4] or PDL-1 [**(D)** *n* = 6] by DC incubated or not for the last 24 h with *F. prausnitzii*, following or not a 45 min-incubation with the CD39 inhibitor Pom1 (10 μM). **(E)** (*n* = 3) LPS-induced IL-12-secretion by DC incubated, or not (Ctrl) with *F. prausnitzii* for 48 h, following or not a 45 min-incubation with the Pom1 inhibitor. **(F,G)**: (*n* = 5) CD39 and PDL-1 expression by DC incubated (F), or not (Ctrl) with *F. prausnitzii* for 24 h, following or not a 45 min-incubation with the IDO1 inhibitor 1-MT. **(A,B):** Wilcoxon test, **(E):** Mann Whitney, **(C,D,F,G)** Paired *t*-test. *p < 0.05, **p < 0.005, ****p < 0.00005.

We then assessed the contribution of the ENTPDase activity of CD39, in the DCF regulatory phenotype. Pre-treatment of DC with the ENTPDase inhibitor Pom1 decreased the secretion of IL-10 (Figure 7A) and IL-27 (Figure 7B), as well as limited the up-regulation of IDO1 and PDL-1 (Figures 7C,D). In contrast, Pom1 did not restore the LPS-induced IL-12 response in DCF (Figure 7E and Supplementary Figures S4G,H). Therefore, CD39 contributes to the secretion of IL-10 and IL-27 by human *F. prausnitzii*-exposed DC, a role previously reported in mouse IL-27-exposed DC (22). Moreover, CD39 up-regulates both IDO1, which has not been reported so far, and PDL-1. The role of IDO1 in the DCF phenotype was then explored using its 1-MT inhibitor. 1-MT completely neutralized the up-regulation of CD39 (Figure 7F) and lowered the expression of PDL-1 (Figure 7G), in DCF. Therefore, IDO1 activity clearly up-regulates CD39 together with IL-27 and, to a limited extent, contributes to PDL-1 expression. Finally, IL-10 and IL-10R inhibition partly restored the LPS-induced expression of CD83 and CD40 (Supplementary Figures S5A,B). In contrast, they did not reinstate the TLR4-mediated IL-12 response (Supplementary Figure S5C), nor altered the secretion of IL-27 (Supplementary Figure S5D).

**Figure 8:**
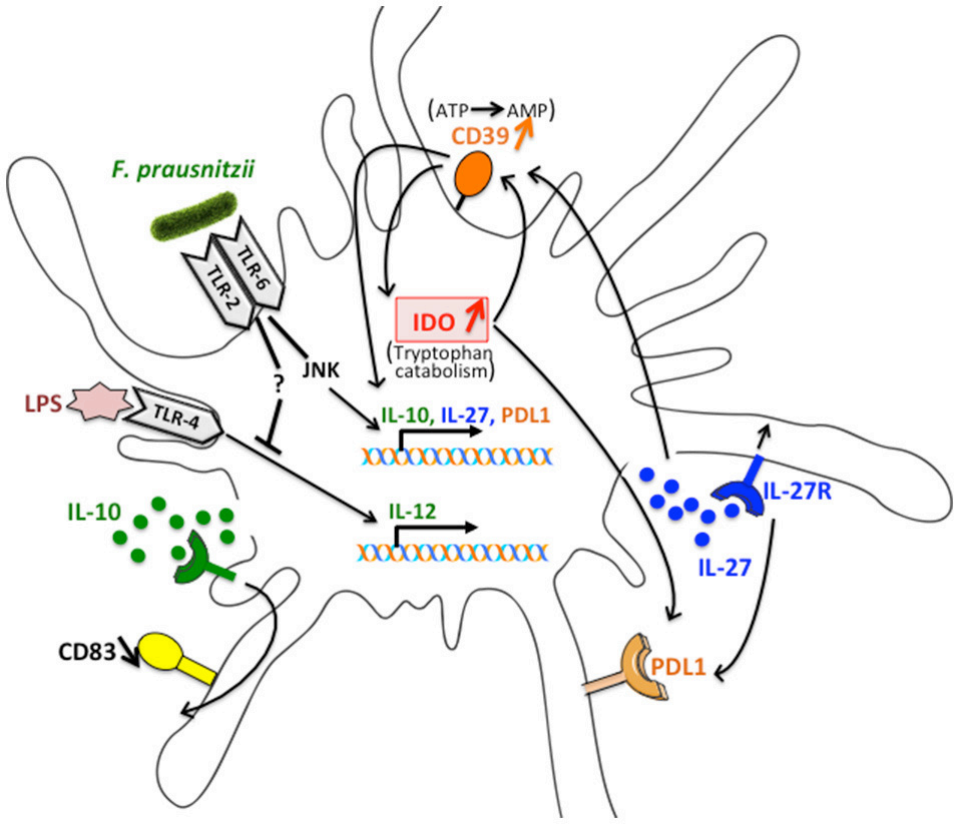
Schematic representation of the interactions involved in the alteration of DC function following exposure to *F. prausnitzii***. **IL-27 secretion induced by *F. prausnitzii* in a JNK dependent manner up-regulates CD39 expression. The ENTPDase activity of CD39 then contributes to IL-10 and IL-27 induction and up-regulates IDO1, in a feed-forward loop. IL-10 secretion also induced via the TLR2/6/JNK pathway limits the LPS-induced expression of CD83, which is affected by TLR2/6 triggering but not JNK blocking. Finally, PDL-1 expression relies on IDO1 enzymatic activity and, partly, on JNK and IL-27 signaling.

Altogether, as summarized (Figure 8), these data suggest that IL-27 secretion induced by *F. prausnitzii* in a JNK-dependent manner, up-regulates CD39 expression. The ENTPDase activity of CD39 then contributes to IL-10 and IL-27 induction, as shown earlier in mouse DC (22), and up-regulates IDO1, in a feed-forward loop. IL-10 secretion also induced via TLR2/6/JNK limits the LPS-induced expression of CD40 and CD83, a role previously reported for Treg-derived IL-10 (32), but does not seem to contribute to IL-12 inhibition, which was affected by TLR2/6 but not JNK blocking. Finally, PDL-1 expression relies on IDO1 enzymatic activity and, partly, on JNK and IL-27 signaling.

## Discussion

Here we show that a single *F. prausnitzii* encounter endowed DC with a unique array of properties that promote Tr1 Treg generation as well as decreased effector lymphocyte development. Accordingly, these DC acquired a potent ability to prime IL-10-secreting T cells, and a decreased capacity to prime IFN-γ- and IL-13-secreting T cells compared to untreated DC. In contrast, DC exposed to the Clostridia M88 and M89 primed IL-13-secreting T cells as control DC did and failed or poorly primed IL-10-secreting T cells, respectively. These data suggest that the regulatory properties of DC, here shown to be induced by *F. prausnitzii*, but less or not by M88 and M89, are involved in the unique ability of this bacterium to induce Tr1-like Treg *in vivo*. Nonetheless, additional studies will be needed to better assess which of these properties are essential to prime Tr1-like colonic Treg.

Supporting the *in vivo* relevance of these *in vitro* results, the presence of *F. prausnitzii*-specific Tr1-like Treg in the human colon indicates that *F. prausnitzii*, or components of it, interacts with antigen presenting cells *in vivo*. Moreover, although human colonic DC remain poorly characterized, some share properties with DCF such as IL-10 secretion (33), an immature phenotype (34), IL-6 secretion and IDO-1 expression (35) as well as a propensity to prime IL-10-secreting T cells *in vitro* (36). In addition, a recent mouse study established the major role of microbiota-induced IL-10 secretion by intestinal APC in gut homeostasis, via promoting Treg generation (37).

We have previously shown that colonic and blood DP8α Treg recognized *F. prausnitzii*, but not the other human *Clostridia* tested, among which M88 and M89 (3, 4), while the latter induced Foxp3 colonic Treg in GF mice (38). This suggests that these bacteria do not participate in human colonic Treg induction. We now show that DC functions were not, or significantly less, altered by M88 and M89 than by *F. prausnitzii*. This result, and potentially also the higher *F. prausnitzii* frequency in the human microbiota (8), may explain the dominant (potentially unique) specificity of human colonic Treg for *F. prausnitzii* (3, 4). Nonetheless, M88 and M89 significantly inhibited TLR4-mediated secretion of pro-inflammatory cytokines IL-12 and TNF-α. Such inhibitions may therefore represent an anti-inflammatory effect shared by many commensals and potentially contribute to a broader anti-inflammatory role of the gut microbiota

The role of IL-10 in inhibiting DC maturation and thereby promoting Tr1-Treg or, together with TGF-β, Foxp3-Treg generation, has been largely documented (11, 12, 16, 17). Interestingly, *F. prausnitzii* systematically induced IL-10 secretion by DC, at variance with M88 and M89. This secretion decreased the levels of co-stimulation molecules on LPS-stimulated DCF. *F. prausnitzii* also differed from M88 and M89 by a systematic induction of IL-27-secreting DC. So far, a physiological role of IL-27 production by DC was shown to prevent autoimmunity in the central nervous system by inhibiting Th17 development (17) and promoting Treg generation, via the induction of CD39 expression (22). However, induction of IL-27 by gut commensals has not been put forward as a mechanism involved in microbiota-induced gut homeostasis, even though one study reported the induction of Tr1 cell differentiation in the mouse colon via this cytokine and IL-10, by the probiotic *Bifidobacterium breve* (25). Our data suggest that IL-27 may contribute to the induction of *F. prausnitzii*-specific Treg in the human colon. In support of this, IL-27 was identified as a candidate gene for IBD susceptibility and a number of studies in IBD mouse models have demonstrated its protective effect (39). However, at a steady state, production of IL-27 by human colon DC has not been reported, although this cytokine was detected in gut tissues during inflammatory processes (40). Interestingly, IL-27 has been shown to be a potent inducer of Tr1 cells, while inhibiting TGF-β-induced Foxp3 Treg development (17). Therefore, both the lack of TGF-β induction and the secretion of IL-27 by *F. prausnitzii*-exposed DC likely explain why human *F. prausnitzii*-specific Treg belong to the Tr1-like subtype, whilst in the mouse colon, induction of TGF-β-secreting DC by *Clostridium* bacteria promotes the development of *Clostridum*-specific Foxp3 colonic Treg (1, 14).

Recognition of commensal ligands by TLR is required for colon injury protection (28). In particular, mouse studies established the role for TLR2 in Treg induction by commensals such as *Bacteroides fragilis* and its component PSA (23), or by *Clostridium*, via TGF-β induction (14). Our neutralization experiments indicate that *F. prausnitzii* induces the secretion of IL-10, by triggering the TLR2/6/JNK pathway, as well as that of IL-27 via JNK activation. In addition, the ATPase activity of CD39 also contributes to the secretion of these regulatory cytokines. To our knowledge this is the first report identifying TLR2/6/JNK and CD39 as mediators of regulatory roles induced by gut commensals. Nonetheless, TLR2/6 was previously shown to promote Tr1 Treg development through induction of IL-10-producing DC, as an escape mechanism used by *Yersinia pestis* (30). Of note, IL-27 production by DC may also be induced by IFN-β (41), AhR activation (42) or DC-SIGN triggering by fucose-containing ligands (43). Our results do not exclude an additional contribution of these inducers to the secretion of IL-27 by DCF. Moreover, the role of TLR2/6 in DC polarization by *F. prausnitzii*, does not exclude contributions of TLR2/2, TLR2/1, NLR or CLR ligands to this process.

We also found that TLR2/6, but not JNK inhibition restored the production of IL-12 chains by DC exposed to *F. prausnitzii*. Therefore, TLR2/6 activation but not JNK signaling is involved in the inhibition of TLR4-mediated IL-12 production in DCF. These data indicate that *F. prausnitzii* activates both TLR2/6/JNK-dependent and TLTR2/6 dependent but JNK-independent pathways.

We could not directly address a role of TLR2/6 in the up-regulation of CD39 and IDO1 expression by *F. prausnitzii* due to non-specific effects of isotypic control Abs. The up-regulation of CD39 in DC by rhIL-27 suggests that *F. prausnitzii*-induced IL-27 contributes to it, and as so would be dependent on JNK signaling. However, JNK inhibition did not affect CD39 up-regulation in DCF, suggesting that it was induced through another mechanism. Accordingly, CD39 up-regulation in DCF was strongly dependent on the enzymatic activity of IDO1. Moreover, the level of IDO1 expression itself was highly dependent on the ATPase activity of CD39, revealing the existence in DCF of a feed-forward loop between these two enzymes.

In conclusion, here we show that *F. prausnitzii*, a *Clostridium* gut bacterium widely recognized as a sensor and a player in human health, exhibits potent tolerogenic abilities, which imprint DC to prime Tr1-like Treg. Moreover, we identified several mediators in this process. These data, together with the presence of Tr1 Treg in the human colon and the decrease, in IBD patients, of both these cells and *F. prausnitzii*, point out to *F. prausnitzii*-induced DC polarization as the first described cellular mechanism whereby the microbiota composition may affect human colon homeostasis. Importantly, Identification of *F. prausnitzii*-induced mediators involved in Treg induction opens therapeutic avenues for the treatment of IBD.

## Author Contributions

JA performed, analyzed, and interpreted experiments as well as contributed to the manuscript. EG contributed to some experiments, the study organization, and discussions. LP allowed for some of the IL-27 experiments. GS contributed to the study organization and discussions. JT contributed to the IL-27-related study. CB provided bacteria. KY allowed for HO-1-related experiments. HS contributed to the manuscript. FA contributed to study design and discussions. FJ supervised the project and wrote the manuscript. EG, FA, JA, HS, and FJ edited the manuscript.

### Conflict of Interest Statement

The authors declare that the research was conducted in the absence of any commercial or financial relationships that could be construed as a potential conflict of interest.
