# Supplementary material for: Faecalibacterium prausnitzii Skews Human DC to Prime IL10-Producing T Cells Through TLR2/6/JNK Signaling and IL-10, IL-27, CD39, and IDO-1 Induction
Source: Front Immunol. 2019 Feb 6;10:143. doi: 10.3389/fimmu.2019.00143 (PMC6373781; doi:10.3389/fimmu.2019.00143)
Supplement: Supplementary file 1 [file Data_Sheet_1.pdf]

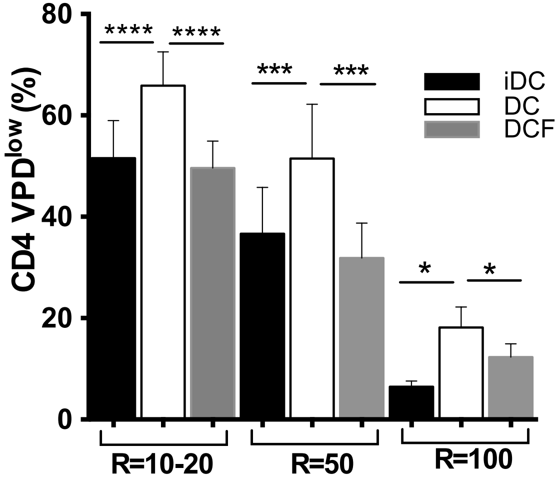

Supplementary figure 1. **DC exposed to *F. prausnitzii* during their differentiation (DCF) have a decreased capacity to stimulate the proliferation of allogeneic CD4 T cells**, **compared to control LPS stimulated DC (DC) and similar to that of immature DC (iDC).** Purifed CD4 T cells were labeled by the violet proliferation dye VPD and stimulated by DC at the indicated lymphocyte/DC ratios (R) (R=10-20: n=17; R=50: n=12, R=100 n=7). The percentage of T cell proliferation was assessed by measuring the fraction of CD4 T cells which diluted the VPD (VPD^{low}) after 10 days of stimulation. Paired t test. P as in Fig 1

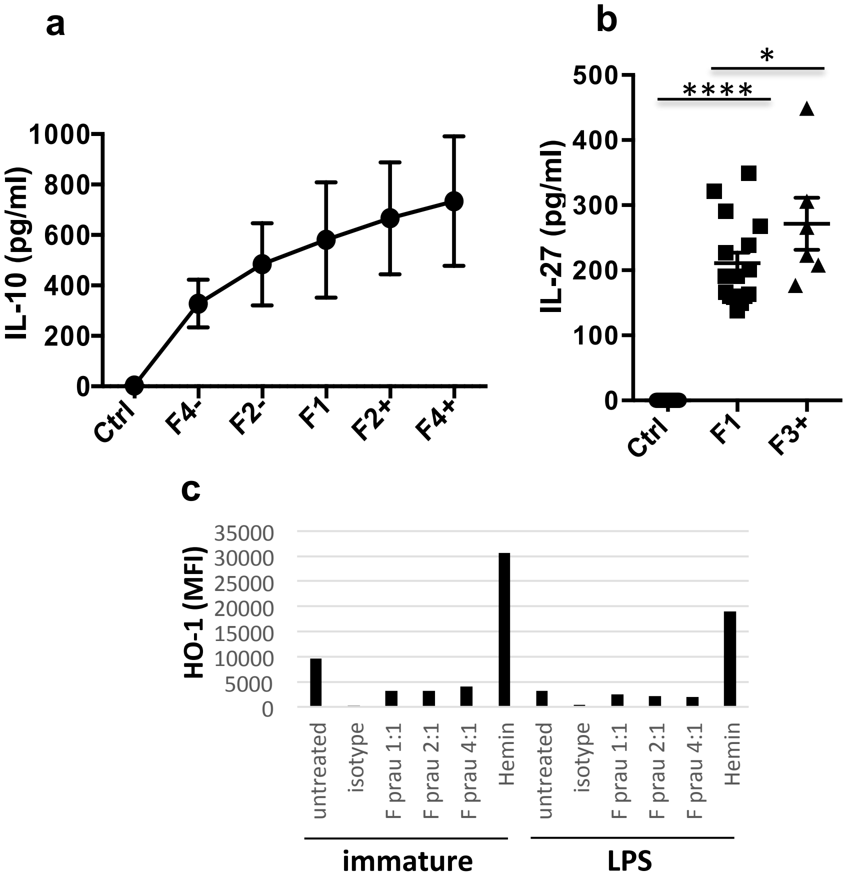

Supplementary figure 2. Effects of *F. prausnitzii* on the expression of immunoregulatory molecules by monocyte-derived DC. **a.** Mean IL-10 levels (n=6) secreted by DC cultured for 24h with GM-CSF and IL-4 and *F.prausnitzii* (n=6); DC/*F. prausnitzii* ratios were 4:1 (F4-) 2:1 (F2-) 1:1 (F1) 1:2 (F2+) or 1:4 (F4+). **b.** IL-27 levels secreted by DC exposed or not (Ctrl) for the last 24h to *F. prausnitzii* at DC:*F. prausnitzii* ratios 1:1 (F1) or 1:3 (F3+). **c.** HO-1 expression measured by flow cytometry in DC exposed or not (untreated) to *F. prausnitzii* at indicated *F. prausnitzii* :DC ratios at the beginning of their differentiation and matured or not by LPS (n=3). Wilcoxon test, p as in Figure 1.

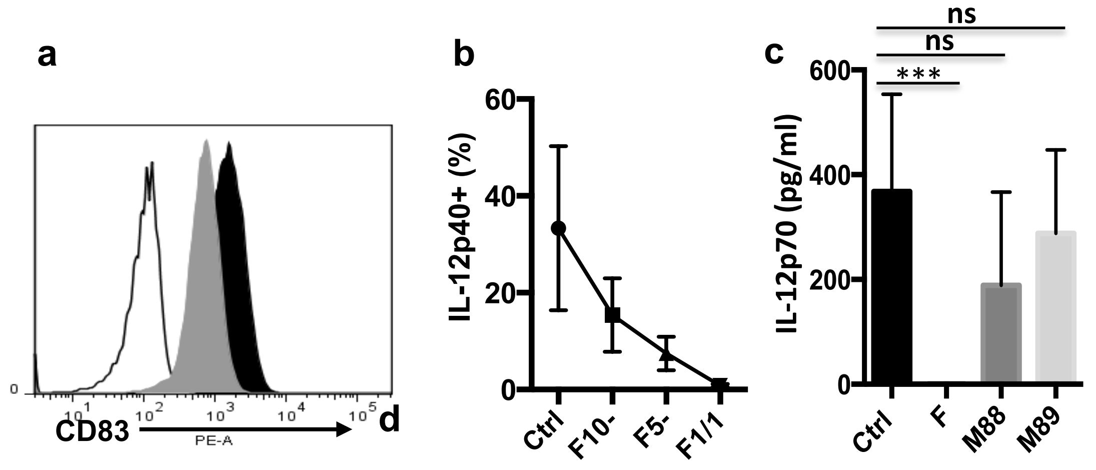

Supplementary figure 3: DC exposure to *F. prausnitzii* inhibits LPS-induced up-regulation of co-stimulation molecules and IL-12 production. a. Representative example of CD83 expression by LPS-matured control DC (black histogram) and DCF (grey histogram), isotype: white histogram. **b.** LPS-induced IL-12p40 expression **(mean % p40$^+$ cells, sem)** by DC from 3 donors exposed or not (ctrl) to *Fprau* at DC:bacterium ratios 1:1 (F1) 5:1 (F5-) or 10:1 (F10-), during the last 48h and then stimulated by LPS for 12h (n=3). **c**. IL-12p70 levels secreted in response to LPS by DC exposed or not to indicated bacteria at the beginning of their differentiation. Wilcoxon, p as in Figure 1.

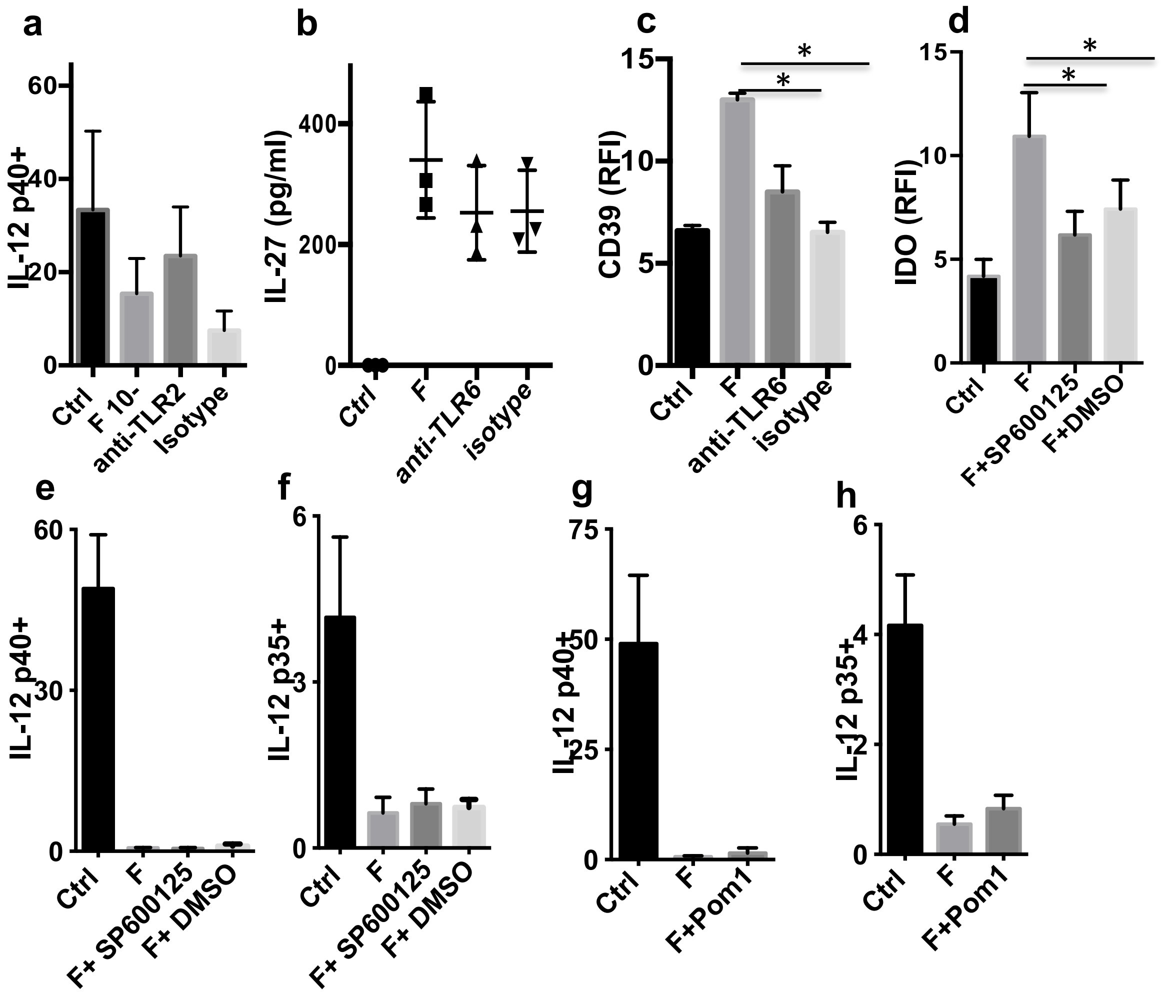

Supplementary figure 4: Mechanisms of DC modulation by *F. prausnitzii*. a. LPS-induced IL-12p40 expression by DC exposed or not (ctrl) for the last 48h to *F. prausnitzii* at DC:bacterium ratio 10:1 (F10-) following or not a treatment with anti-TLR-2 antibody or its isotype control (n=3). **b.** IL-27 secretion by DC exposed or not (ctrl) for the last 48h to *F. prausnitzii*, following or not a treatment by the anti-TLR-6 antibody or its isotype control. **c.** CD39 expression (n=6) by DC exposed or not to *F. prausnitzii* for the last 48h, following or not treatment by the anti-TLR-6 antibody or its isotype control **d.** IDO1 expression by DC exposed or not (ctrl) to *F. prausnitzii* for the last 48h, following or not a treatment with the JNK inhibitor SP600125 or its DMSO vehicle (n=4). **e-h**: LPS or LPS+R848-induced IL-12p40 or p35 expression by DC exposed (F) or not (ctrl) for the last 48h to *F. prausnitzii,* following or not treatment by indicated inhibitors (n=3). Paired t test.

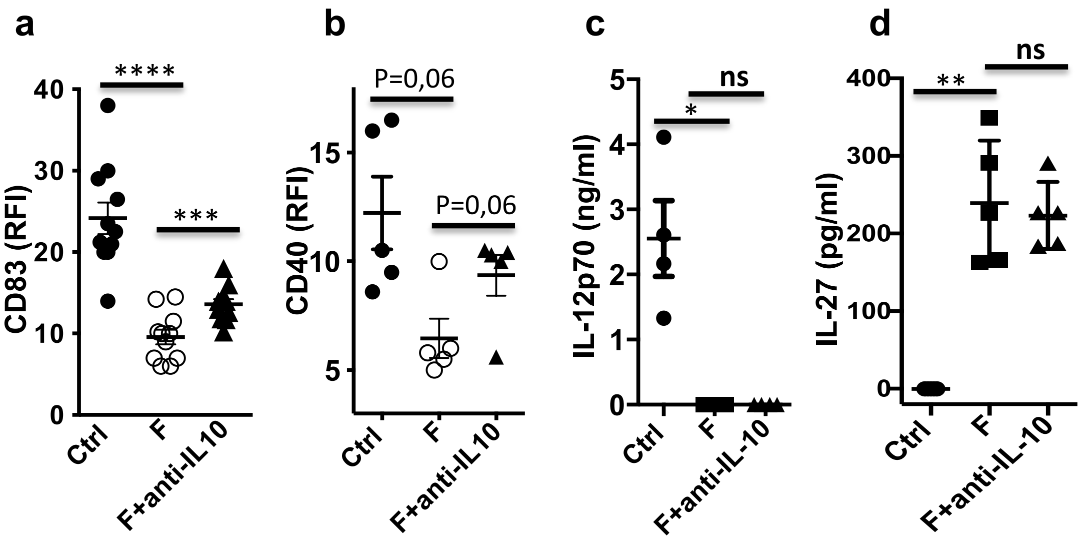

Supplementary figure 5 : IL-10-dependent DC modulation. CD83 (**a,** n=11) and CD40 (**b,** n=5) expression by DC exposed or not to *F. prausnitzii* at the beginning of their differentiation, following or not pre-incubation with IL-10 and IL-10R neutralizing Ab and then stimulated by LPS during 48h. IL-12 p70 (**c** n=4) and IL-27 (**d,** n=5) secretion by DC exposed or not to *F. prausnitzii* for the last 48h in the presence or not of neutralizing anti-IL-10 and anti IL-10R antibodies (n=4). Paired t test.
